# Supplementary material for: Non-Laboratory-Based Risk Prediction Tools for Undiagnosed Pre-Diabetes: A Systematic Review
Source: Diagnostics (Basel). 2023 Mar 29;13(7):1294. doi: 10.3390/diagnostics13071294 (PMC10093270; doi:10.3390/diagnostics13071294)
Supplement: Supplementary file 1 [file diagnostics-13-01294-s001.zip › diagnostics-2309855-supplementary.pdf]

Supplementary Material

Table S1. Search strategy

Search terms used for MEDLINE and Embase via Ovid <1946 to March Week 2 2022>

1	PREDIABETES/
2	prediab$.tw,kf,ot.
3	(pre adj2 diab$).tw,kf,ot.
4	(pre adj2 NIDDM).tw,kf,ot.
5	exp HYPERGLYC?EMIA/
6	((elevat$ or high$) adj2 glucose).tw,kf,ot.
7	((impair$ or dysfunct$) adj2 glucose tolera$).tw,kf,ot.
8	((impair$ or dysfunct$) adj2 glucose regulat$).tw,kf,ot.
9	(borderline$ adj2 diabet$).tw,kf,ot.
10	(impair$ adj2 glyc?em$).tw,kf,ot.
11	(insulin adj2 resist$).tw,kf,ot.
12	IGT.tw,kf,ot.
13	IFG.tw,kf,ot.
14	IGR.tw,kf,ot.
15	(undiagno$ adj2 diabet$).tw,kf,ot.
16	(pre adj2 T2DM).tw,kf,ot.
17	1 or 2 or 3 or 4 or 5 or 6 or 7 or 8 or 9 or 10 or 11 or 12 or 13 or 14 or 15 or 16
18	(risk adj2 asses$).tw,kf,ot.
19	(risk adj2 screen$).tw,kf,ot.
20	(risk adj2 scor$).tw,kf,ot.
21	(risk adj2 ident$).tw,kf,ot.
22	(risk adj2 detect$).tw,kf,ot.
23	(risk adj2 predict$).tw,kf,ot.
24	(risk adj2 categor$).tw,kf,ot.
25	(risk adj2 stratif$).tw,kf,ot.
26	(early adj2 diagno$).tw,kf,ot.
27	18 or 19 or 20 or 21 or 22 or 23 or 24 or 25 or 26
28	tool$.tw,kf,ot.
29	rule$.tw,kf,ot.
30	algorith$.tw,kf,ot.
31	((math$ or statisti$) adj2 (model$ or equation$)).tw,kf,ot.
32	calculat$.tw,kf,ot.
33	MACHINE LEARNING/
34	multivaria$.tw,kf,ot.
35	(log$ adj2 (model$ or regress$)).tw,kf,ot.
36	(classif$ adj2 tree$).tw,kf,ot.
37	(decision$ adj2 analy$).tw,kf,ot.
38	28 or 29 or 30 or 31 or 32 or 33 or 34 or 35 or 36 or 37
39	17 and 27 and 38
40	non-diabet$.tw.
41	39 not 40
42	limit 41 to humans
43	limit 42 to english language

Search terms used for CINAHL via EBSCOhost

prediabetes or impaired glucose tolerance or impaired fasting glucose or prediabetic state or early stage diabetes or borderline diabetes hyperglycaemia or hyperglycemia or high blood sugar or high blood glucose

AND

risk assess* or risk screen* or risk scor* or risk identif* or risk detect* or risk predict* or early diagnos* or risk categor* or risk stratif*

AND

tool or rule or algorithm or score* or statistic* model or equation or calculator or machine learning or multivaria* regress* or classif* tree or decision analysis or support vector machine

Search terms used for PubMed

((prediabetes[Title/Abstract] OR impaired glucose tolerance[Title/Abstract] OR impaired fasting glucose[Title/Abstract] OR prediabetic state[Title/Abstract] OR early stage diabetes[Title/Abstract] OR borderline diabetes hyperglycaemia[Title/Abstract] OR hyperglycemia[Title/Abstract] OR high blood sugar[Title/Abstract] OR high blood glucose[Title/Abstract]) AND (risk assess*[Title/Abstract] OR risk screen*[Title/Abstract] OR risk scor*[Title/Abstract] OR risk identif*[Title/Abstract] OR risk detect*[Title/Abstract] OR risk predict*[Title/Abstract] OR early diagnos*[Title/Abstract] OR risk categor*[Title/Abstract] OR risk stratif*[Title/Abstract])) AND (tool[Title/Abstract] OR rule[Title/Abstract] OR algorithm[Title/Abstract] OR score*[Title/Abstract] OR statistic* model[Title/Abstract] OR equation[Title/Abstract] OR calculator[Title/Abstract] OR machine learning[Title/Abstract] OR multivaria* regress*[Title/Abstract] OR classif* tree[Title/Abstract] OR decision analysis[Title/Abstract] OR support vector machine[Title/Abstract])

Supplementary Material

Table S2. Result post-appraisal by Critical Appraisal Skills Programme (CASP) Clinical Prediction Rule Checklist

	Q1.	Q2.	Q3.	Q4.	Q5.	Q6.	Q7.	Q8.	Q9.	Q10.	Q11.
Abbas, 2021	+	+	−	+	+	+	+	−	−	+	+
Bahijri, 2020	+	+	−	+	+	+	+	−	−	+	+
Barengo, 2017	+	+	−	+	+	+	+	+	−	+	+
Dong, 2022	+	+	−	+	+	+	+	+	+	+	+
Fu, 2014	+	+	+	+	+	+	?	−	+	+	+
Fujiati, 2017	+	+	+	+	+	+	+	−	−	+	+
Gao, 2010	+	+	+	+	+	+	?	+	+	+	+
Gray, 2010	+	+	+	+	+	+	+	+	−	+	+
Gray, 2012	+	+	+	+	+	+	+	+	−	+	+
Gray, 2013	+	+	+	+	+	+	+	−	−	+	+
Handlos, 2013	+	+	−	+	+	+	?	+	−	+	+
Hische, 2010	+	+	+	+	+	+	?	+	−	+	+
Koopman, 2008	+	+	+	+	+	+	+	+	−	+	+
Memish, 2015	+	+	−	+	+	+	?	−	−	+	+
Rajput, 2019	+	+	−	+	+	+	+	−	−	+	+
Robinson, 2011	+	+	−	+	+	+	+	−	−	+	+
Sadek, 2022	+	+	+	+	+	−	+	−	−	+	+
Stiglic, 2018	+	+	−	+	+	+	+	+	−	+	+
Tan, 2016	+	+	+	+	+	+	?	+	−	+	+
Wang, 2015	+	+	+	+	+	+	+	−	+	+	+
Xin, 2010	+	+	−	+	+	+	+	−	−	+	+
Yu, 2010	+	+	−	+	+	+	+	−	−	+	+

Note: “+” refers to “yes”, “-” refers to “no”, “?” refers to “can’t tell” on the CASP checklist
